# Prognostic significance of HER3 in patients with malignant solid tumors

**DOI:** 10.18632/oncotarget.18007

**Published:** 2017-05-18

**Authors:** Qin Li, RuiXue Zhang, Han Yan, PengFei Zhao, Li Wu, Hui Wang, Teng Li, Bangwei Cao

**Affiliations:** ^1^ Department of Oncology, Beijing Friendship Hospital, Capital Medical University, Beijing 100050, China; ^2^ Department of Internal Medicine, The First Hospital, Tsinghua University, Beijing 100016, China; ^3^ Department of Health Statistics, School of Public Health, Shanxi Medical University, Taiyuan 030001, China; ^4^ Beijing Key Laboratory for Precancerous Lesion of Digestive Diseases, Beijing Friendship Hospital, Capital Medical University, Beijing 100050, China; ^5^ Beijing Digestive Diseases Center, Beijing Friendship Hospital, Capital Medical University, Beijing 100050, China

**Keywords:** HER3, malignant tumors, prognosis, molecular markers, immunohistochemistry

## Abstract

Human epidermal growth factor receptor 3 (HER3) is closely involved in tumor progression and is an important target of therapy. To evaluate the prognostic significance of HER3 in malignant solid tumors, we searched the PUBMED, EMBASE and CNKI databases for relevant studies written in English or Chinese up to December 2015. Fifteen studies comprising 2964 patients were identified. The HER3^+^ rate ranged from 9.0-75.1 % in malignant solid tumors: 30.3-75.1 % in breast cancers, 51.1-74.5 % in colorectal cancers, 13.7-59.0 % in gastric cancers, and 54.5-74.4 % in cervical cancers. For patients with a malignant solid tumor, the death risk was higher for those with a HER3^+^ tumor than for those with a HER3^−^ tumor (HR 1.60, 95% CI: 1.27 - 2.02, *P* < 0.001). Subgroup analysis revealed this was also the case for patients with digestive or gastric cancer (HR 1.78, *P* < 0.001; HR 2.18, *P* < 0.001). By contrast, HER3 had no prognostic significance in colorectal or breast cancer (HR 1.52, *P* = 0.296; HR 1.23, *P* = 0.108). HER3^+^ is thus associated with poor survival in overall and in gastric cancer. The prognostic significance of HER3^+^ in other tumors is uncertain and deserves further study.

## INTRODUCTION

Human epidermal growth factor receptor 3 (HER3, also known as erbB-3) is a distinctive member of the HER family, as it lacks certain residues that are essential for catalytic activity of other kinases. The function of HER3 was once considered to be passive and the clinical value of HER3 was greatly underestimated. However, in recent years, biochemical analysis confirmed that the kinase domain of HER3 was always “active” in the sense that it had a C-lobe that was competent to engage and activate the kinase domains of the other members of HER family [1]. Shi F's study also revealed that the intracellular region of HER3 was capable of binding ATP and promoting auto-phosphorylation [2]. The heterodimerization of HER3 with HER1/HER2/HER4 triggers the activation of signaling network, especially phosphatidylinositol 3-kinase/protein kinase B and downstream molecular, which is implicated in tumorigenesis, proliferation, migration and metastasis [3, 4].

With the deep understanding of the structure and function of HER3, the researches about the HER3 are carried out in full swing. HER3 has been verified to promote the tumor progression and metastasis [5, 6]. The activation of HER3/phosphatidylinositol 3-kinase/protein kinase B signal pathway has led the targeted resistance in non-small-cell lung cancer, breast cancer and other tumors [7–10]. So much attention has focused on the strategies to inhibit the activity of HER3. The human HER3 monoclonal antibody KTN3379 inhibited tumor growth in divergent tumor models driven by either ligand-dependent or independent mechanisms *in vitro* and *in vivo* [11]. The novel anti-HER3 antibody patritumab abrogates cetuximab resistance mediated by heregulin in colorectal cancer cell [10]. The bispecific antibody MM-111 forms a trimeric complex with HER2 and HER3, effectively inhibiting the HER2/HER3 oncogenic unit and heregulin-induced HER3 activation, showing antitumor activity [12]. The results of phase I study about anti-HER3 monoclonal antibody lumretuzumab and HER3/EGFR antibody MEHD7945A showed their good tolerance and the clinical benefits in patients with advanced cancer [13, 14].

In addition to the clinical development of anti-HER3 therapies, the predictive and prognostic significance of HER3 over-expression in malignant solid tumors is also the focus of clinical attention, but the research findings are contradictory. Some studies found that the positive presence of HER3 (HER3^+^) was associated with worse prognosis [15–17], whereas others drew the opposite conclusions [18, 19]. Therefore, the present systematic analysis was performed to assess the prognostic significance of HER3^+^ in patients with malignant solid tumors.

## RESULTS

### Selection of the trials

**Figure 1:**
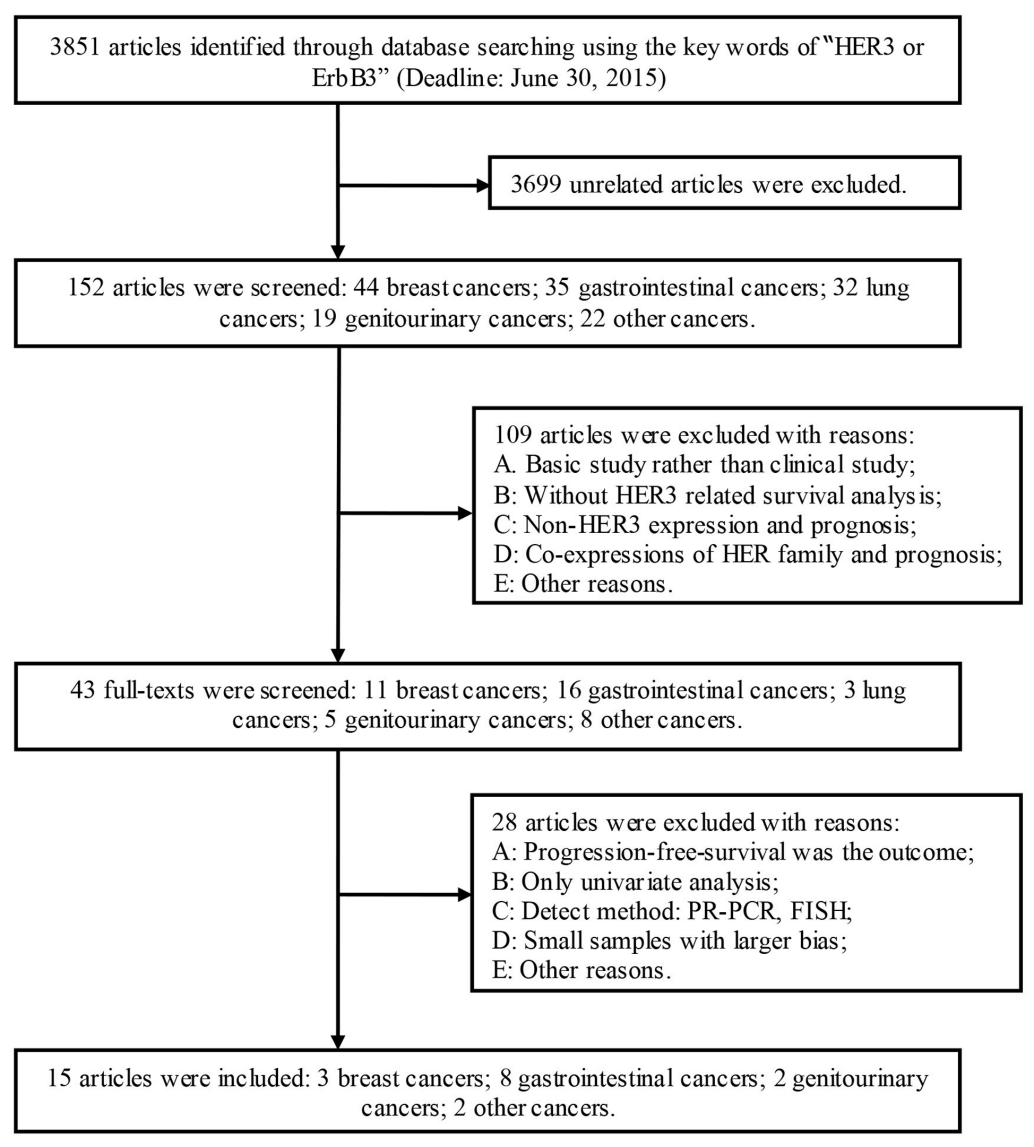
Schematic of the study selection

In accordance with the search strategy (Figure 1), initially 3851 articles were considered. Screening of the primary title led to the exclusion of 3699 articles. In the remaining 152 articles, 109 articles were excluded for the following reasons: they were basic research rather than clinical study; there was no HER3-related survival analysis; or for other reasons. The full texts of the remaining 43 articles were screened, and 28 articles were excluded for the following reasons: the observed outcome was progression-free survival; the article contained only the result of univariate analysis; the method of detecting HER3 was reverse transcription polymerase chain reaction or fluorescence in situ hybridization; smaller samples led to a larger bias; or for other reasons. Finally, 15 articles were included (Table 1) [15–18, 20–30]. The PRISMA checklist is shown in Supplementary Table 1.

**Table 1:** Characteristics of the eligible studies included in the systematic assessment *

First authors	Year	NOS	Tumor	*n*	Follow-up	HER3^+^, %	IHC antibody
Bae SY	2013	9	Breast cancer	950	109.7m(0.6-198.1)	56.0	M7297, Dako, mouse monoclonal
Park YH	2014	9	Breast cancer	109	36.0m(12.0-72.5)	30.3	M7297, Dako, mouse monoclonal
Sassen A	2008	9	Breast cancer	173	125.6m	75.1	5A12, NanoTools
Baiocchi G	2009	8	Colorectal cancer	109	57.4m(2.0-165.8)	69.7	RB-9211, Lab Vision, epitope specific rabbit antibody
Beji A	2011	7	Colorectal cancer	110	Not available	74.5	C-17, Santa Cruz
Lédel F	2014	8	Colorectal cancer	236	Not available	71.6	SP71, Abcam, rabbit monoclonal
Scartozzi M	2012	7	Colorectal cancer	90	Not available	51.1	DAK-H3-IC, Dako, mouse monoclonal
Hayashi M	2008	9	Gastric cancer	134	1943d(50-3197)	59.0	Mouse monoclonal antibody, Neomarkers
Li G	2013	8	Gastric cancer	161	39.6m	55.9	Mouse antibody, Shanghai Longisland Biotec
Zhang XL	2009	8	Gastric cancer	102	8.0-30.0m	13.7	RTJ1, Beijing Zhongshan Jinqiao Biotechnology
Hirakawa T	2011	8	Pancreatic cancer	126	24.1m(1.0-138.0)	41.3	Antihuman HER3 mouse monoclonal, Nanotools
Fuchs I	2007	8	Cervical carcinoma	78	60.0m(1.0-180.0)	74.4	Not mention
Lee CM	2005	7	Cervical carcinoma	55	24.0m(1.0-227.0)	54.5	MS-725-P, NeoMarkers
Reschke M	2008	8	Melanoma	217	31.0-81.0m	55.3	C-17, Santa Cruz.
Takikita M	2011	8	Head and neck squamous cell carcinoma	378	1.0-180.0m	9.0	RTJ.2, Santa Cruz, mouse monoclonal.

* Detection is IHC in all cases

### Main characteristics of the included studies

**Figure 2:**
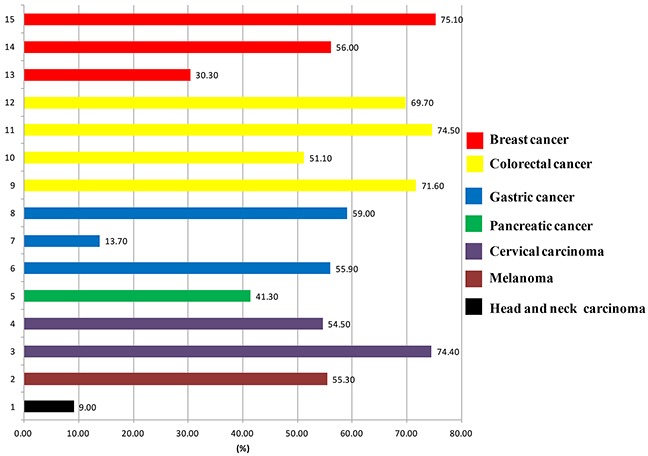
The positive rates of HER3 in different malignant solid tumors

A total of 2964 patients were included in the assessment (Table 1). There were 1168 patients with breast cancer, 545 with colorectal cancer, 397 with gastric cancer, 126 with pancreatic cancer, 378 with head and neck squamous cell carcinoma, 217 with melanoma, and 133 with cervical carcinoma. The rates of HER3^+^ were 30.3 - 75.1% in breast cancers, 51.1 - 74.5% in colorectal cancers, 13.7 - 59.0% in gastric cancers, 54.5 - 74.4% in cervical carcinomas, 53.3% in melanomas, and 9.0% in head and neck squamous cell carcinomas (Figure 2). According to the Newcastle-Ottawa Scale (NOS) for quality, the scores of all the articles were 6 - 9 stars. The reagents used in the Immunohistochemistry (IHC) for HER3 and the definition of HER3^+^ were described in Tables 1 and 2.

**Table 2:** Definition of HER3 positive expression among the studies

Authors	Tumor	subgroup	Definition of HER3 positive expression
Bae SY	Breast	I	HER3 staining was categorized by criteria A*. Negative = scores 0; Positive = scores 1 + scores 2+ and 3+.
Park YH	Breast	I	HER3 staining scored according to criteria A*. Negative = scores 0; Positive = scores 1 + scores 2+ and 3+.
Sassen A	Breast	I	HER3 staining scored according to criteria A*. Negative = scores 0; Positive = scores 1 + scores 2+ and 3+.
Takikita M	Head and neck squamous cell carcinoma	I	HER3 staining scored according to criteria A*. Negative = scores 0; Positive = scores 1 + scores 2+ and 3+.
Beji A	Colorectal	II	HER3 staining was categorized by criteria A*. Negative = scores 0 and 1+; Positive = scores 2+ and 3+.
Lédel F	Colorectal	II	HER3 staining was categorized by criteria A*. Negative = scores 0 and 1+; Positive = scores 2+ and 3+.
Hayashi M	Gastric	II	HER3 staining was categorized by criteria A*. Negative = scores 0 and 1+; Positive = scores 2+ and 3+.
Li G	Gastric	II	HER3 staining was categorized by criteria A*. Negative = scores 0 and 1+; Positive = scores 2+ and 3+.
Zhang XL	Gastric	II	HER3 staining was categorized by criteria A*. Negative = scores 0 and 1+; Positive = scores 2+ and 3+.
Hirakawa T	Pancreatic	II	HER3 staining was categorized by criteria A*. Negative = scores 0 and 1+; Positive = scores 2+ and 3+.
Lee CM	Cervical	II	HER3 negative = scores 0 and 1+; HER3 positive: = scores 2+ and 3+.
Fuchs I	Cervical	III	HER3 negative ≤3; HER3 positive > 3. Score = positive cells (0, negative; 1, 1-10%; 2, 11%-50%; 3, 51%-80%; 4, >80%) × staining intensity (0-3).
Reschke M	Melanoma	III	HER3 negative ≤ 6; HER3 positive > 6. Score = positive cells (0, negative; 1, 1-10%; 2, 11%-50%; 3, 51%-80%; 4, >80%) × staining intensity (0-3).
Scartozzi M	Colorectal	III	HER3 negative ≤ 8; HER3 positive > 8. Score = positive cells (0, <10%; 1, 10-25%; 2, 26-50%; 3, 51-75%; 4, >75%) × staining intensity (0-3).
Baiocchi G	Colorectal	IV	HER3 negative ≤ 1.5; HER3 positive 1.5-3. Score = [The cytoplasmic staining(0-3)+The membranous staining(0-3)]/the numbers of tumor cores evaluated.

* Criteria A. HER3 staining was categorized by intensity as 0, 1+, 2+, and 3+. 0, samples with no staining at all, or in < 10 % of the tumor cells; 1+, a faint or barely perceptible incomplete staining in >10 % of tumor cells; 2+, weak-to-moderate staining in >10 % of tumor cells; 3+, strong staining in >10-30 % of tumor cells.

### The overall analysis of HER3^+^ and survival time

**Figure 3:**
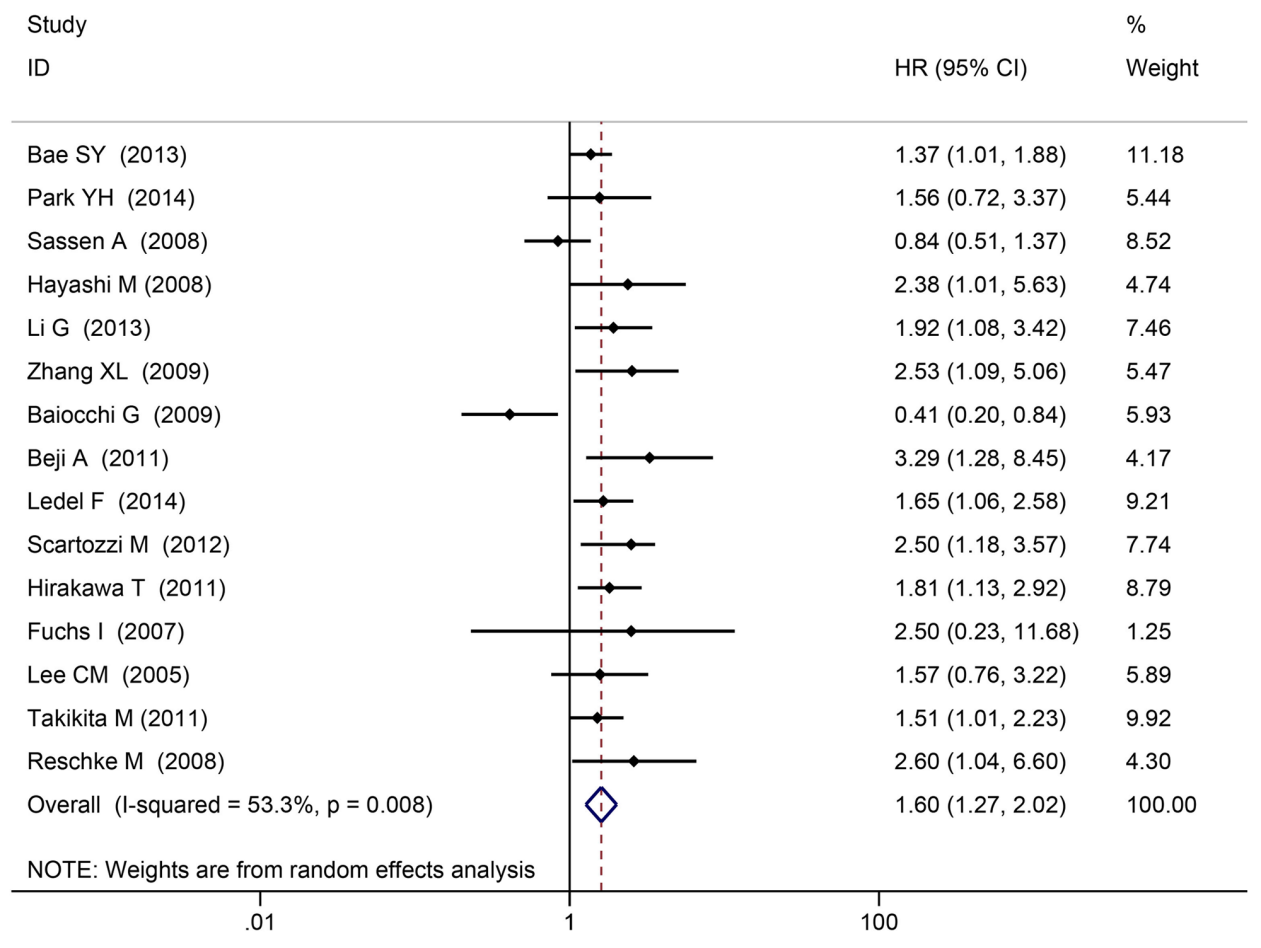
The overall analysis of HER3^+^ and survival time

**Figure 4:**
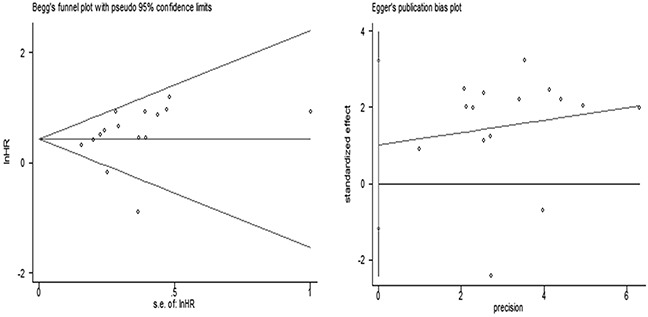
The Begg's test and Egger's test for the overall analysis

Fifteen articles reported the Hazard ratios (HRs) of HER3^+^ predicting overall survival (OS), gained through multivariate analysis. There was significant heterogeneity among the overall studies (*P* = 0.008), so the random-effects model was applied. Considering HER3^+^ status as a risk factor predicting death, the risk of death in HER3^+^ patients was 1.60-fold than that of HER3^−^ patients (HR 1.60, 95% Confidence intervals (CIs): 1.27 - 2.02, *P* < 0.001) (Figure 3). No significant publication bias was determined by the Begg's test or Egger's test (*P* = 0.092, *P* = 0.337, respectively; Figure 4).

In the sensitivity analysis, after removing one study with a large population [Bae SY 2013, n = 816], the risk of death in HER3^+^ patients was 1.78-fold than that of HER3^−^ patients (*P* < 0.001). When a series of sensitivity analyses were conducted by deleting one set of data each time, no significant differences in the final results were observed.

The sub-analysis was done according to the diagnostic criteria and cutoff values of HER3 positive expression (Supplementary Figure 1). The risk of death in HER3^+^ patients was 1.30-fold than that of HER3^−^ patients in subgroup I (95%CI: 1.05 – 1.61, *P* < 0.001); The risk of death in HER3^+^ patients was 1.91-fold than that of HER3^−^ patients in subgroup II (95%CI: 1.52 - 2.41, *P* < 0.001); The risk of death in HER3^+^ patients was 2.52 -fold than that of HER3^−^ patients in subgroup III (95%CI: 1.59 – 4.01, *P* < 0.001).

### Subgroup analysis of HER3^+^ and survival time

#### Breast cancer

**Figure 5:**
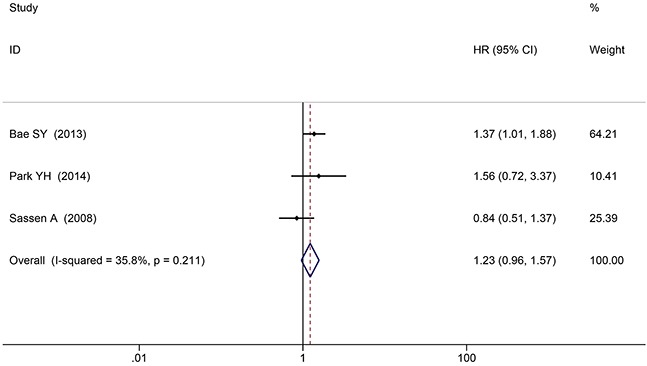
Breast cancer subgroup analysis of HER3^+^ and survival time

Three articles concerned breast cancer. There was no significant heterogeneity among the studies (*P* = 0.211), so the fixed-effects model was applied. The HER3^+^ was not a risk factor predicting death in patients with breast cancer (HR 1.23, 95%CI: 0.96 - 1.57, *P* = 0.108; Figure 5). No significant publication bias was determined by Begg's test or Egger's test (both *P* > 0.05).

#### Digestive tumors

**Figure 6:**
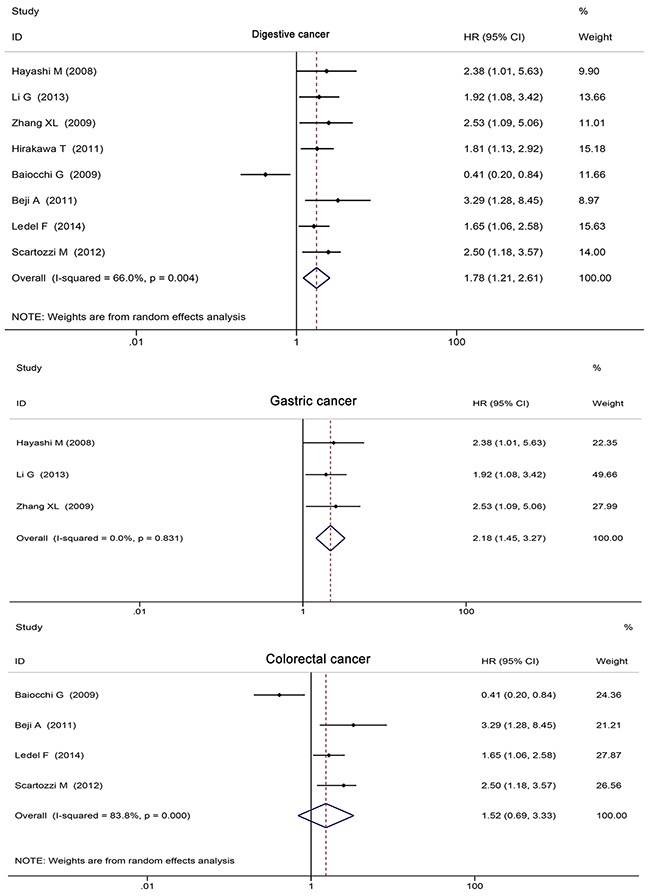
Digestive tumors subgroup analysis of HER3^+^ and survival

Eight articles concerned digestive tumors (4 colorectal, 3 gastric, and 1 pancreatic cancer). There was significant heterogeneity among these studies (*P* < 0.001), so the random-effects model was applied. Merged effects values showed that the risk of death in HER3^+^ patients was 1.78-fold than that of HER3^−^ patients (HR 1.78, 95%CI: 1.21 - 2.61, *P* < 0.001; Figure 6). No significant publication bias was determined by Begg's test and Egger's test (both *P* > 0.05).

The gastric cancer subgroup analysis suggested that HER3^+^ status was an excellent predictive risk factor for death (HR 2.18, 95%CI: 1.45 - 3.27, *P* < 0.001; Figure 5). No significant publication bias was determined by Begg's test and Egger's test (both *P* > 0.05).

The colorectal subgroup analysis showed that HER3^+^ status was not a predictive risk factor for death (HR 1.52, 95%CI: 0.69 - 3.33, *P* = 0.296; Figure 5). No significant publication bias was determined by Begg's test and Egger's test (both *P* > 0.05).

## DISCUSSION

The systematic analysis showed that HER3 positive expression was associated with worse OS in patients with malignant solid tumors, the risk of death in HER3^+^ patients was 1.60-fold than that of HER3^−^ patients in the overall analysis (*P* < 0.001). The systematic analysis of Ocana A [31] drew similar conclusions by way of different statistical indicators, i.e., HER3^+^ was associated with worse OS at both 3 years and 5 years. Our study analyzed the data obtained by unified detection method and the HRs calculated through multivariate analysis, and our study had a larger sample of patients and a longer follow-up time, therefore, the result was more convincing. Our result was supported by the sensitivity analyses, which confirmed the high stability and reliability of the overall research. Subgroup analysis according to diagnostic criteria and cutoff value obtained the similar conclusion with the overall analysis.

Breast cancer is a highly heterogeneous disease [32] and the prognostic value of HER3 seems to be uncertain. Previous studies reported that HER3^+^ was associated with established prognostic indicators, such as higher histological grading [33] and primary breast tumors larger than 2 cm [34]. Giltnane JM found that the high-HER3 group had a 53% 10-year survival compared to 69% in the low-HER3 group (*P* < 0.001) [35]. Berghoff AS reported HER3-overexpression was not correlated with OS, time to brain metastases in the whole population, but negative correlation was observed in the HER2-positive subgroup population [36]. However, Sassen A reported the expression status of HER3 was not associated with survival time [20]. In 2015, Hellenic Cooperative Oncology Group validation study reported that the combination of high EGFR, high HER2, low HER3 and low HER4 mRNA expression was associated with a trend for shorter OS and significantly worse disease-free survival in high-risk early breast cancer patients [37]. Knowlden JM reported that HER3mRNA expression was associated with improved OS as well as estrogen receptor, which is a favorable prognostic factor for breast cancer [38]. In our subgroup analysis of breast cancer, the HER3^+^ rates was 30 - 75%, the risk of death in the HER3^+^ group was not significantly higher than that of the HER3^−^ group.

In our systematic analysis of digestive tumor, the risk of death in HER3^+^ patients was significantly increased than that of HER3^−^ patients (*P*<0.001). The HER2/HER3 heterodimer is considered the most active oncogenic unit, and HER3 is a crucial factor in HER2-mediated tumor cell growth and proliferation [39]. HER3 seems to have the similar prognostic value as HER2. Wang S's meta-analysis showed that HER2^+^ status was related to poor prognosis of gastric cancer (HR 1.58, *P* < 0.001) [40]. Begnami et al.'s study showed that both HER2 and HER3 are predictors of poor outcome in gastric carcinomas [41]. In our gastric cancer subgroup analysis, HER3^+^ status was associated with the poor outcome (HR 2.18, *P* < 0.001).

There is almost no HER3 expression in normal colon tissue. However, the HER3^+^ rate in colorectal cancer tissue is up to 51-75%. Conclusions regarding the value of HER3 for predicting clinical outcome of colorectal cancer were contradictory [18, 22, 42]. Kapitanovic S reported that the median survival time of patients with HER3^−^ (181.1 wk) was significantly longer than that of patients with HER3^+^ (113.9 wk) [42]. In Beji A's study, the strong presence of membranous HER3 indicated a higher risk of tumor-associated death (HR 3.29, *P*< 0.05), establishing HER3 as a putative novel independent prognostic marker for colorectal cancer [22]. In wild-type Kirsten Ras (KRAS) colorectal cancer patients treated with cetuximab, the median progression-free survival and OS were 6.3 and 13.6 months in the HER3^−^ group, 2.8 and 10.5 months in the HER3^+^ group, HER3^+^ seemed to be a negative prognostic factor in wild-type KRAS colorectal cancer patients, the combined analysis of HER3 and KRAS might be an effective strategy for better selection of responding colorectal cancer patients [23]. Lédel F reported HER3^+^ was an independent negative prognostic factor for OS in the entire population of colorectal cancer patients and in the subgroup with colon cancer stage II, but not in stage III [16], it indicated that HER3^+^ status was not strong as a prognostic factor, and the prognostic value decreased when dividing the patients into subgroups. He later reported that HER3^+^ status correlated to shorter disease-free survival in the patients with distal colon cancer (HR 0.56, *P*< 0.05) [43]. On the contrary, Baiocchi G's results showed that HER3^−^ status was an independent prognostic factor for lower survival; the 5-year survival rates were 51.5% in HER3^−^ patients and 77.6% in HER3^+^ patients [18]. In our present colorectal cancer subgroup analysis, HER3^+^ was not significantly associated with the OS (HR 1.52, *P* = 0.296).

We obtained positive result from the whole analysis of malignant solid tumor and subgroup analysis of digestive tumor and gastric cancer. However, the sub-analysis of breast cancer and colorectal cancer didn't show the expected positive conclusions. Summing up the characteristics in subgroup, the differences in clinical staging, sample sizes, IHC antibody used and others might prevent investigators from reaching definitive conclusions. Of course, the differences of HER2 status and hormone receptor status in breast cancer, lesion sites and KRAS status in colorectal cancer, make the related subgroup analysis very challenging. Further hierarchical analysis was needed.

In all included articles, only the results obtained by IHC and multivariate analysis were using for systematic analysis. Although reverse-transcription polymerase chain reaction, fluorescence in situ hybridization, and VeraTag have relatively higher sensitivity and specificity, but these testing methods are not be routinely applied in clinical practice [44–48], and the mixed analysis of HER3 expression detected by different methods might bring more bias, therefore, only the results of IHC were considered. To avoid the bias caused by numerous clinical factors, studies reporting HRs for HER3 predicting OS via multivariate analysis were included. Despite all the efforts, the analysis still has limitations. First, all included articles are defined as high quality with NOS of 7 – 9, however, the literature-based analysis is compromised by the potential for publication bias. Second, there is no standardized method and consensus diagnostic threshold to evaluate HER3 expression currently. Therefore, the substantial heterogeneity could not be fully compensated by applying the random-effects model. An internationally standardized diagnostic method is urgently needed. However, IHC is the most feasible and most reliable method for assessing the HER3 expression.

HER3^+^ is associated with the poor survival in the overall analysis and gastric cancer subgroup analysis, however, the prognostic significance of it in other tumors are uncertain and deserves further study. However, interactions among ligand, HER3, HER family members and downstream signaling molecules are intricate, the predicting significance of HER-related signaling molecular co-expression pattern should be taken into account. The inherent diversity and complexity of each tumor maybe influence the precise of the result, and stratified studies should be encouraged. Of course, unified detection method and reagent, and standardized diagnosis criteria will provide the best support for determining the prognostic value of HER3 positive expression in malignant solid tumors.

## MATERIALS AND METHODS

### Literature search strategy

The systematic analysis was performed in accordance with the criteria of the Preferred Reporting Items for Systematic Reviews and Meta-analyses (PRISMA) [49]. The databases PubMed, Embase and CNKI were searched for articles reporting the prognostic significance of HER3. Original articles written in English or Chinese and published on or before 30 Dec 2015 were collected.

To ensure that all relevant articles were reviewed, the references of articles on associations between members of the epidermal growth factor receptor family and prognosis were manually screened. The initial search used the MeSH terms: “HER3 OR erbB 3 OR Receptor, erbB 3 OR erbB-3 Receptor OR c-erbB-3 Protein OR c erbB 3 Protein OR erbB-3 Protein OR erbB 3 Protein” AND “Neoplasm OR Neoplasia OR Tumor OR Tumors OR Cancer OR Cancers”.

### Inclusion criteria and category

All the articles reporting the HRs for HER3^+^ predicting overall survival using multivariate analysis were included in the systematic assessment. All the articles detected HER3 via IHC. The diagnostic criteria and cut-off of HER3^+^ expression were depicted and summarized in Table 2. The diagnostic criteria were divided into three categories, and the cutoff values were divided into four categories. (1). HER3^+^ was categorized by staining intensity as 0, 1+, 2+, and 3+. 0, samples with no staining at all, or in < 10 % of the tumor cells; 1+, a faint or barely perceptible incomplete staining in >10 % of tumor cells; 2+, weak-to-moderate staining in >10 % of tumor cells; 3+, strong staining in >10-30 % of tumor cells. One cutoff value was “Negative = scores 0; Positive = scores 1 + scores 2+ and 3+”; another cutoff value was “Negative = scores 0 and 1+; Positive = scores 2+ and 3+”. (2). HER3^+^ was categorized by “the percentages of positive cells × staining intensity”. (3). HER3^+^ was categorized by “(the cytoplasmic staining + the membranous staining)/the numbers of tumor cores evaluated”. The patients were classified into subgroup I, II, III, IV according to the diagnostic criteria and the cutoff values. (Table 2)

### Data extraction

All likely abstracts were assessed independently by two investigators (Qin Li and Peng-fei Zhao) based on the predefined inclusion criteria. If only one investigator considered an abstract eligible, the full text of the article was retrieved and reviewed in detail by both investigators. Any discrepancy was resolved by an arbiter (Rui-xue Zhang) or by contacting the authors of the original article.

The survival data and HRs were extracted from original articles. The following were recorded: authors’ names, journal title, year of publication, tumor types, follow-up time, antibody used for the detection, the HER3 examination method and scoring protocol, number and ratio of HER3^+^, and the cutoff value for defining HER3^+^ (Table 1 and Table 2).

### Assessment of methodological quality

The quality of the studies was assessed independently by two researchers (Qin Li and Peng-fei Zhao), using the NOS (http://www.ohri.ca/programs/clinical_epidemiology/oxford.asp). In these observational studies, the assessment included selection of cases, comparability of the cohorts with regard to design or analysis, and outcomes. Studies with a total NOS score of 5 - 9 were defined as high quality, whereas a score 0 - 4 was defined as low quality.

### Statistical analysis

The statistical analyses were carried out using Stata version 11.0 (StataCorp LP, Texas, USA). Heterogeneity was investigated using Cochrane's Q-test and I^2^ statistics. *P* > 0.1 and I^2^ < 50% was considered lack of heterogeneity among the studies, and the pooled estimation of HR for each study was calculated according to the fixed-effects model (Mantel-Haenszel method). *P* < 0.1 and I^2^ > 50% indicated that the studies were heterogeneous, and then the random-effects model (DerSimonian-Laird method) was applied. The HRs was the principal measures of effect and was presented with 95% CIs. All reported *P* values were from two-sided versions of the respective tests. *P* < 0.05 was considered statistically significant. Sensitivity analyses were also conducted, the changes of the combined effects were observed by excluding studies with large samples or by removing a set of research data one at a time. Publication and selection biases were investigated through funnel plots based on Egger's and Begg's tests [50, 51].

## SUPPLEMENTARY MATERIALS FIGURES AND TABLES





## Abbreviations

HER3: Human epidermal growth factor receptor 3
NOS: Newcastle-Ottawa Scale
IHC: Immunohistochemistry
HRs: Hazard ratios
OS: Overall survival
CIs: Confidence intervals
KRAS: Wild-type Kirsten Ras
