# Supplementary material for: Prognostic significance of HER3 in patients with malignant solid tumors
Source: Oncotarget. 2017 May 18;8(40):67140–51. doi: 10.18632/oncotarget.18007 (PMC5620162; doi:10.18632/oncotarget.18007)
Supplement: Supplementary file 1 [file oncotarget-08-67140-s001.pdf]

Prognostic significance of HER3 in patients with malignant solid tumors

SUPPLEMENTARY FIGURE AND TABLE

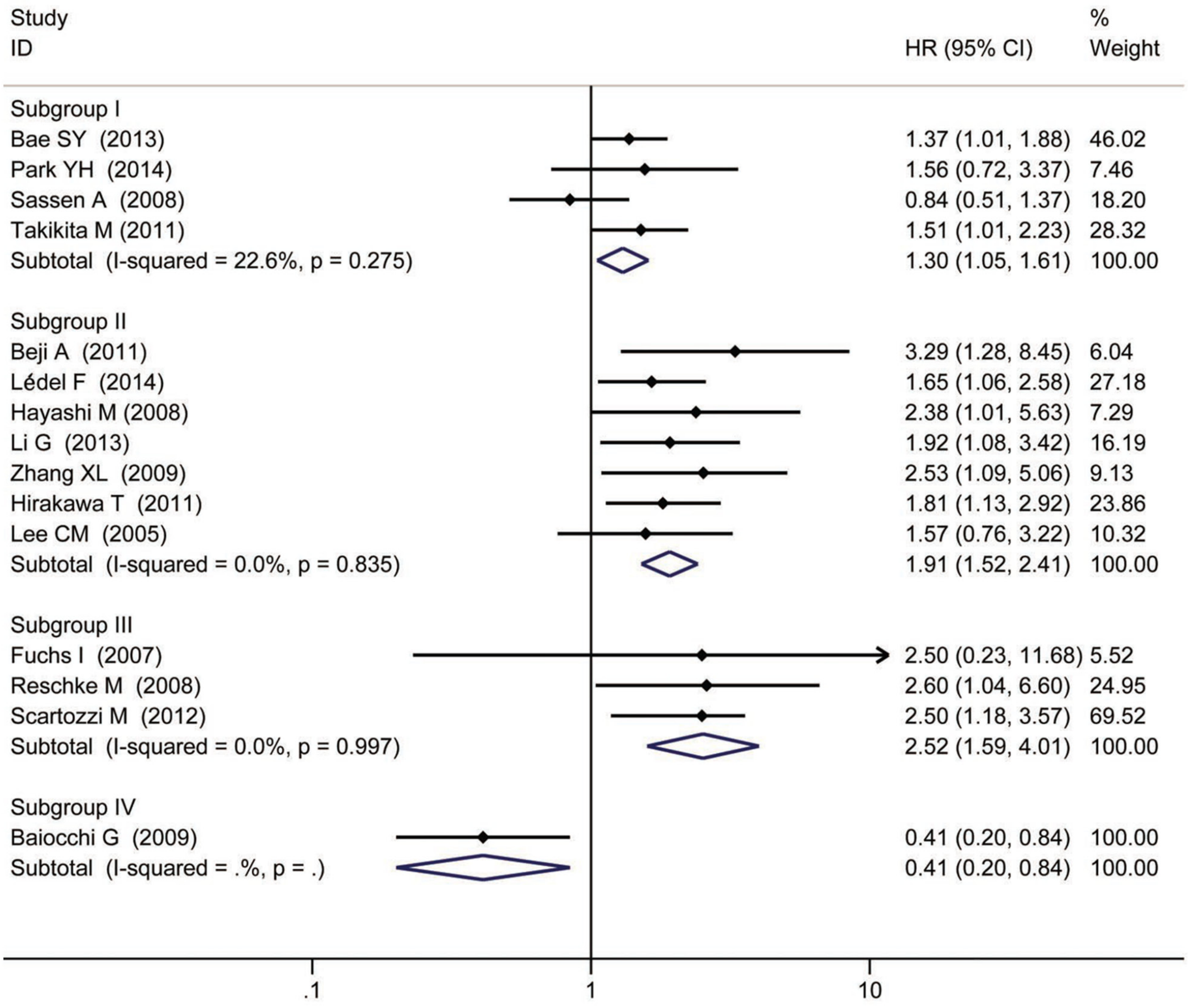

Supplementary Figure 1: The subgroup analysis according to the diagnostic criteria of HER3 positive expression.

Supplementary Table 1: PRISMA 2009 checklist.

See Supplementary File 1
